# Evaluation of the 2022 West Nile virus forecasting challenge, USA

**DOI:** 10.1186/s13071-025-06767-2

**Published:** 2025-04-23

**Authors:** Ryan D. Harp, Karen M. Holcomb, Renata Retkute, Alisa Prusokiene, Augustinas Prusokas, Zeynep Ertem, Marco Ajelli, Allisandra G. Kummer, Maria Litvinova, Stefano Merler, Ana Pastore y Piontti, Piero Poletti, Alessandro Vespignani, Andre B. B. Wilke, Agnese Zardini, Kelly Helm Smith, Philip Armstrong, Nicholas DeFelice, Alexander Keyel, John Shepard, Rebecca Smith, Andrew Tyre, John Humphreys, Lee W. Cohnstaedt, Saman Hosseini, Caterina Scoglio, Morgan E. Gorris, Martha Barnard, S. Kane Moser, Julie A. Spencer, Maggie S. J. McCarter, Christopher Lee, Melissa S. Nolan, Christopher M. Barker, J. Erin Staples, Randall J. Nett, Michael A. Johansson

**Affiliations:** 1 Division of Vector-Borne Diseases, Centers for Disease Control and Prevention, Fort Collins, CO USA; 2 Global Systems Laboratory, National Oceanic and Atmospheric Administration, Boulder, CO USA; 3 Cooperative Programs for the Advancement of Earth System Science, University Corporation for Atmospheric Research, Boulder, CO USA; 4 Department of Plant Sciences, University of Cambridge, Cambridge, UK; 5 School of Natural and Environmental Sciences, Newcastle University, Newcastle upon Tyne, UK; 6 Independent Researcher, London, UK; 7 School of Systems Science and Industrial Engineering, Binghamton University, State University of New York, Binghamton, NY USA; 8 Laboratory for Computational Epidemiology and Public Health, Department of Epidemiology and Biostatistics, Indiana University School of Public Health, Bloomington, IN USA; 9 Department of Epidemiology and Biostatistics, Indiana University School of Public Health, Bloomington, IN USA; 10 Center for Health Emergencies, Fondazione Bruno Kessler, Trento, Italy; 11 Laboratory for the Modeling of Biological and Socio-Technical Systems, Network Science Institute, Northeastern University, Boston, MA USA; 12 National Drought Mitigation Center, University of Nebraska-Lincoln, Lincoln, NE USA; 13 Connecticut Agricultural Experiment Station, New Haven, CT USA; 14 Icahn School of Medicine at Mount Sinai, New York, NY USA; 15 Division of Infectious Diseases, Wadsworth Center, New York State Department of Health, Albany, NY USA; 16 Department of Atmospheric and Environmental Sciences, University at Albany, State University of New York, Albany, NY USA; 17 Department of Pathobiology, University of Illinois at Urbana‐Champaign, Champaign, IL USA; 18 Regulatory Science, Bayer AG, Chesterfield, MO USA; 19 Foreign Animal Diseases Research Unit, Agricultural Research Service, National Bio- and Agro-Defense Facility, U.S. Department of Agriculture, Manhattan, KS USA; 20 Foreign Arthropod-Borne Animal Diseases Research Unit, Agricultural Research Service, National Bio- and Agro-Defense Facility, U.S. Department of Agriculture, Manhattan, KS USA; 21 Department of Electrical and Computer Engineering, Kansas State University, Manhattan, KS USA; 22 Information Systems and Modeling, Los Alamos National Laboratory, Los Alamos, NM USA; 23 Division of Biostatistics, University of Minnesota, Minneapolis, MN USA; 24 Genomics and Bioanalytics, Los Alamos National Laboratory, Los Alamos, NM USA; 25 Department of Epidemiology and Biostatistics, University of South Carolina, Columbia, SC USA; 26 Department of Computer Science and Engineering, University of South Carolina, Columbia, SC USA; 27 Department of Pathology, Microbiology, and Immunology, School of Veterinary Medicine, University of California, Davis, CA USA; 28 Division of Vector-Borne Diseases, Centers for Disease Control and Prevention, San Juan, PR USA

**Keywords:** West Nile virus, West Nile virus neuroinvasive disease, Forecasting, Vector-borne disease, Ensemble, Weighted interval scoring, Logarithmic scoring, Multi-model assessment

## Abstract

**Background:**

West Nile virus (WNV) is the most common cause of mosquito-borne disease in the continental USA, with an average of ~1200 severe, neuroinvasive cases reported annually from 2005 to 2021 (range 386–2873). Despite this burden, efforts to forecast WNV disease to inform public health measures to reduce disease incidence have had limited success. Here, we analyze forecasts submitted to the 2022 WNV Forecasting Challenge, a follow-up to the 2020 WNV Forecasting Challenge.

**Methods:**

Forecasting teams submitted probabilistic forecasts of annual West Nile virus neuroinvasive disease (WNND) cases for each county in the continental USA for the 2022 WNV season. We assessed the skill of team-specific forecasts, baseline forecasts, and an ensemble created from team-specific forecasts. We then characterized the impact of model characteristics and county-specific contextual factors (e.g., population) on forecast skill.

**Results:**

Ensemble forecasts for 2022 anticipated a season at or below median long-term WNND incidence for nearly all (> 99%) counties. More counties reported higher case numbers than anticipated by the ensemble forecast median, but national caseload (826) was well below the 10-year median (1386). Forecast skill was highest for the ensemble forecast, though the historical negative binomial baseline model and several team-submitted forecasts had similar forecast skill. Forecasts utilizing regression-based frameworks tended to have more skill than those that did not and models using climate, mosquito surveillance, demographic, or avian data had less skill than those that did not, potentially due to overfitting. County-contextual analysis showed strong relationships with the number of years that WNND had been reported and permutation entropy (historical variability). Evaluations based on weighted interval score and logarithmic scoring metrics produced similar results.

**Conclusions:**

The relative success of the ensemble forecast, the best forecast for 2022, suggests potential gains in community ability to forecast WNV, an improvement from the 2020 Challenge. Similar to the previous challenge, however, our results indicate that skill was still limited with general underprediction despite a relative low incidence year. Potential opportunities for improvement include refining mechanistic approaches, integrating additional data sources, and considering different approaches for areas with and without previous cases.

**Graphical Abstract:**

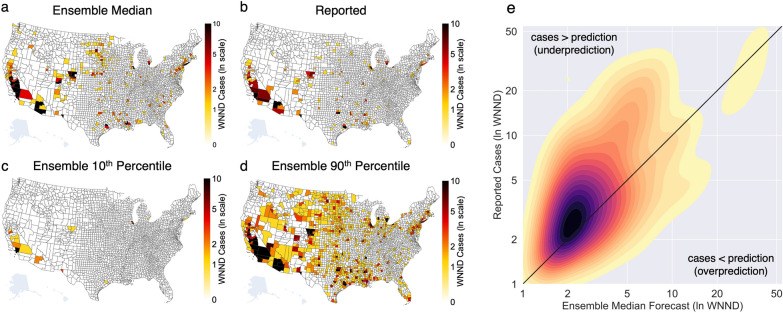

**Supplementary Information:**

The online version contains supplementary material available at 10.1186/s13071-025-06767-2.

## Background

West Nile virus (WNV) is the most impactful mosquito-borne pathogen in the continental USA. WNV was introduced to the USA in 1999 [1] and became endemic with a median of 1288 neuroinvasive cases reported annually from 2005 to 2022. WNV is predominately vectored by *Culex* mosquitoes and amplified in passerine birds (e.g., songbirds) [2–4], with humans infected incidentally. While most people who become infected with WNV are asymptomatic (~75–80%; [5, 6]) or quickly recover from a febrile illness, approximately 1 in 150 develop a severe, neuroinvasive form of the disease (West Nile virus neuroinvasive disease; WNND) and approximately 10% of WNND cases are fatal [7, 8].

WNV has become a persistent health threat in the USA, with substantial variability in incidence across the country and over time. For example, U.S. WNND burden jumped from under 500 cases (2011) to nearly 3000 cases (2012) the following year. Single counties can exhibit a similar dramatic variability; Maricopa County, AZ reported 956 WNND cases in 2021, nearly 5× its previous annual maximum and more than 20× its historical median [9]. This variability both motivates and complicates efforts to forecast WNV. Anticipation of WNV outbreaks may allow for more effective and targeted preventive actions such as vector control, promotion of personal protection measures, and healthcare provider alerts that could be appropriately timed and scaled to maximize their potential impact. However, despite the impact of WNV and extensive research to understand the ecology of WNV transmission, few predictive models have been used to guide public-health actions, and no effective nationwide forecast exists [10]. To address this deficiency, the U.S. Centers for Disease Control and Prevention (CDC) and Council for State and Territorial Epidemiologists have hosted a series of open, collaborative WNV forecasting challenges beginning in 2020 [11]. Similar challenges have been implemented to assess real-time forecasting capabilities for influenza and coronavirus disease 2019 (COVID-19) [12] and have resulted in reliable, routinely produced short-term ensemble forecasts providing situational awareness for trends in these respiratory diseases, though it should be noted that inherent differences in data availability and predictability between respiratory diseases and WNV exist.

In the initial WNV Forecasting Challenge, which aimed to predict the annual WNND cases reported per county in 2020, Holcomb et al. [11] found that no model outperformed one solely informed by historical WNND data. This forecast predicted the number of cases as a negative binomial distribution fitted to the numbers previously observed in each county; this simple model performed similarly to, or outperformed, more sophisticated models that included climate or mosquito variables, as well as an ensemble forecast. When comparing forecast methods, inclusion of factors such as climate, demographics, and mosquito distributions were associated with relative improvements in performance. Despite the positive impact of including these factors, no model outperformed the simple historical model, potentially due to the inherent difficulties in characterizing the influence of these factors within a single forecast model, appropriately calibrating county-specific baselines, or model overfitting. Moreover, the simple forecast included location-specific information but not year-specific information, thus by definition it cannot provide insight on whether a county is likely to experience a particularly bad year for WNV—the kind of forecast that would have the greatest potential benefit. Thus, properly determining why the simple historical model outperforms more complex models is important to advancing WNV forecast skill and motivates our work here. Lastly, Holcomb et al. revealed potential place-based opportunities for forecast improvement, specifically in counties with large populations, high interannual variability in WNND cases, and relatively warm or cold (i.e., not moderate) extreme winter temperatures. Here, building off the insights revealed in the 2020 Challenge, we present analysis and findings of the 2022 WNV Forecasting Challenge, again assessing forecast skill and identifying factors associated with variation in forecast skill.

## Methods

### Organization

Teams were invited to participate in the open 2022 WNV Forecasting Challenge by the CDC Epidemic Prediction Initiative through widely distributed emails and postings starting in February 2022. Participating teams were provided with annual counts of WNND for all 3108 counties in the contiguous USA and Washington, D.C. from 1999 to 2021 from ArboNET, the CDC-managed national arboviral disease surveillance system. The 2021 data were provisional at the time of the challenge but are now finalized and publicly available [13]. Teams could use any modeling approach and any additional data sources (e.g., climate or human demographic data) to assist their modeling efforts (complete information on team modeling approaches can be found in the supplementary materials; Text S1). Additional details about the organization and administration of the Challenge are available on the project GitHub repository [14].

### Forecasts

Modeling teams submitted probabilistic forecasts of the number of WNND cases to be reported to ArboNET in each county in the contiguous USA and Washington, D.C. for all of 2022. We chose WNND as the forecast target over all cases of WNV (i.e., including non-neuroinvasive diseases cases) as WNND cases are most likely to be properly diagnosed and consistently reported due to the severity of the disease (~1 in 150 WNV cases; [7]). For each county, forecasts were provided in a quantile-based format for 23 prediction intervals: 1%, 2.5%, 5% to 95% at 5% intervals, 97.5%, and 99%, which allowed for greater forecast-specificity compared with the 2020 Challenge, wherein teams forecast the likelihood of annual county caseload falling within predefined discrete bins (e.g., 0, 1–5, 6–10, …, > 200). Given the typical seasonality of WNV with a peak in late summer, initial forecasts were submitted in advance of an 30 April 2022 deadline to simulate providing an actionable amount of time to implement public health responses given an accurate WNV forecast, though additional optional forecasts could be submitted monthly by the end May, June, or July.

In addition to the forecasts submitted by participating teams, we created two baseline forecasts and an ensemble forecast. Similar to the 2020 Challenge, the baseline forecasts were based entirely on historical WNND case counts. First, we created a “naive” historical model by fitting a single universal negative binomial distribution to all county-year counts of WNND from 2000 to 2021. This model provides the same forecast for each county and, in effect, presumed all counties have the same underlying probability distribution of WNND cases regardless of population or other county-specific characteristics. Second, we created a county-specific historical negative binomial model by independently fitting a negative binomial distribution to annual WNND cases for each county from 2000 to 2021. This model did not include any temporal information but did capture county-specific heterogeneity in historical cases. Finally, we created an ensemble model to produce a forecast leveraging the combined model forecasts. This ensemble model was derived from all submitted forecast models, as well as the historical negative binomial baseline model, by calculating the median values of the suite of forecasts at each quantile. For example, the 95th percentile of the ensemble forecast was generated by taking the median value of all 95th percentile values in the suite of forecasts, and so on. We refer to this model as the “ensemble model” or “ensemble forecast” for the remainder of the text.

### Evaluation

We used two proper score metrics [15] to evaluate the skill of submitted and baseline WNV forecasts: the weighted interval score (WIS) and the logarithmic score. As noted above, the 2022 Challenge used a different forecast format (quantiles) than the 2020 Challenge (probability bins), removing the need to decide bins a priori and therefore enabling forecast models to more directly characterize uncertainty. WIS can be calculated directly on quantile forecasts [16] and can be decomposed to provide additional information about forecast dispersion and bias, and is therefore the primary focus of our analyses. We calculated WIS as described in Bracher et al. [16] with one additional step: we first log-transformed both the forecasts and the observations (adding one to the case count prior to transforming) to reduce the correlation between higher valued forecasts and higher WIS values [17]. We further calculated the components of WIS to assess dispersion, underprediction, and overprediction, where dispersion characterizes the width of the probabilistic prediction intervals and under- and overprediction characterize directional forecast bias.

We also included logarithmic scores to assess potential differences between the two scoring metrics and for continuity with the 2020 Challenge (see Text S2 for complete description of calculating logarithmic scores from quantiles). We characterized overall forecast skill for each team as the mean of all county-level scores and applied a non-parametric bootstrapping approach to compare the statistical significance of differences between all pairs of nationwide model-specific scores, as the full complement of county-level scores were not normally distributed (see Text S3 for complete description of bootstrapping methodology).

### Model component regression modeling

In addition to examining individual model performance, we evaluated the relationship of forecast skill (as measured by WIS) with model characteristics. Similar to the 2020 Challenge evaluation, we examined both model frameworks (e.g., ensemble, Bayesian components) and model inputs (e.g., climate, mosquito surveillance, demographic, land use data) in an effort to identify any methodological traits that may be consistent across well-performing forecast models. For this, we performed a Bayesian generalized linear regression with backward selection using the stan_glm function of the rstanarm package [18] in R (v4.4; [19]). See supporting information for a complete list of variables considered (Text S4; Table S1).

### County-level contextual regression modeling

We also investigated the impact of county-specific factors on ensemble model forecast skill to identify place-based factors that were associated with better or worse forecast skill (i.e., predictability). To do so, we examined a wide range of potential input variables related to environmental factors, human demographics, and historical WNND case incidence. This analysis can inform future forecast model development by identifying the characteristics of locations that are currently hard to predict and the bounds of predictability inherent to particular locations. We fitted Bayesian generalized additive models using the stan_gamm4 function in the rstanarm package in R [18, 19]. See supporting information for the complete list of variables considered and extended methodology (Text S5).

## Results

In total, eight teams participated in the 2022 WNV Forecasting Challenge (see Text S1 for affiliations and additional team details); six of the eight teams submitted updated forecasts after April, however, overall change and differences in rank were limited in later submissions, despite some forecasts showing improved or decreased skill (Figure S1). We therefore focused our scoring analysis on forecasts submitted in April, the only date when all teams submitted a forecast.

**Fig. 1:**
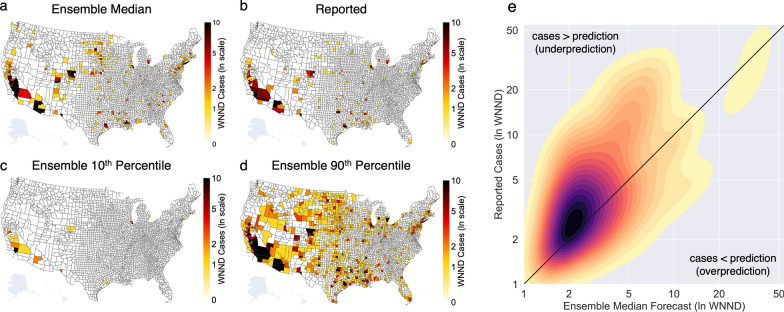
2022 WNND Burden and Ensemble Forecasts. **a** Ensemble forecast median for reported WNND cases in 2022 for all counties in the contiguous USA **b** Number of WNND cases reported to CDC via ArboNET in 2022. **c** The ensemble forecast 10th percentile and **d** 90th percentile. **e** Kernel density estimate of the median number of WNND cases predicted by the ensemble forecast (*x*-axis) and reported cases (*y*-axis) where zero values have been omitted for visualization (yellow–red–dark purple fill for increasing density of points). Black diagonal line illustrates a hypothetical perfect forecast line. Fill to the upper-left of the diagonal line shows density of instances where the ensemble median forecast underpredicted caseload while fill to the bottom-right of the diagonal line shows overpredicted caseload

The ensemble forecast median was equal to the 10-year median for 93% of counties, 98% of which had a median of zero cases over the 10 years. Of the remaining counties, 6% and less than 1% had ensemble forecast medians lower or higher, respectively, than the 10-year median. Despite the county-level overlap in the ensemble forecast and 10-year medians, the sum of ensemble forecast medians across all counties was 425 WNND cases, well below the 10-year national median of 1386, indicative of the right skew in historical distributions. In 2022, 826 WNND cases were reported in the USA, fewer cases than the 10-year median and more cases than the sum of median forecasts for 2022. Only 8% of counties reported WNND cases greater than their 10-year historical median and 13 counties reported their first-ever case of WNND (~1% of the 1054 counties that had not reported a case of WNND previously). Overall, the ensemble forecast tended to underpredict reported incidence (Fig. 1).

**Fig. 2:**
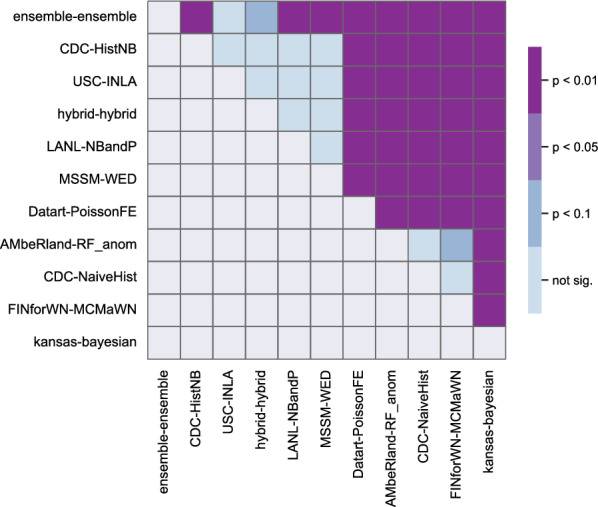
Comparison of mean model scores. Level of statistical significance of differences in model performance for each pair of models (blue–purple shading). Comparison performed using WIS for all counties using bootstrapping methodology (see Text S3 for details)

**Fig. 3:**
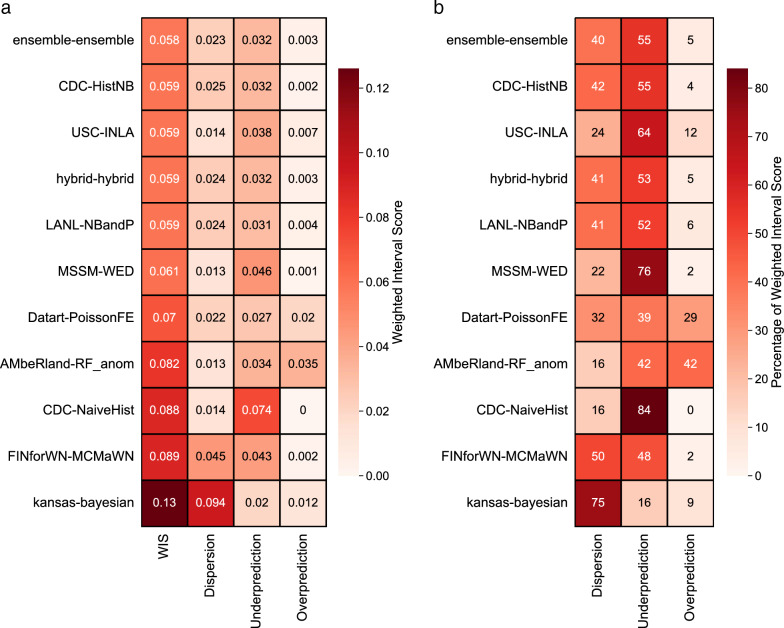
Weighted interval score component heatmaps. **a** Heatmap of weighted interval score and components: dispersion, underprediction, and overprediction. Values for each component-model combination are shown and shaded. For all, larger values indicate worse skill. **b** Same as (a) but the percentage of each component’s contribution to the total score (e.g., a 50 for dispersion indicates that 50% of the total WIS is attributable to the dispersion component)

An examination of team-specific forecast skill revealed that the ensemble model had a higher average skill than all submitted and baseline models (WIS = 0.058, 9 of 10 comparison *P*-values < 0.05; Fig. 2 and Table 1). The historical negative binomial model—the highest performing model in the 2020 WNV Forecasting Challenge [10]—had the second highest skill (WIS = 0.059), though its performance was statistically indistinguishable from four other models within the second tier of forecast performance; these models were all statistically different from the remaining five models (all pairwise comparison *P*-values < 0.01). We found differences in the model-specific skill reflected in the components of the WIS (Fig. 3). Most models were biased toward underprediction (53% of the total WIS on average), though dispersion (36%) and overprediction (11%) substantively contributed toward total WIS as well. While most individual models followed this pattern, three models exhibited different patterns of bias or dispersion (Fig. 3): the largest WIS component for both the FINforWN-MCMaWN and Kansas–Bayesian forecasts was dispersion, while the highest component for the AMbeRland-RandomForest_anomaly forecast was overprediction (exceeding underprediction by ~0.5%).

**Table 1:** All county mean model scores. Mean weighted interval scores across all counties in the contiguous USA, ordered by increasing WIS (i.e., worse skill) across all counties

Model	All county WIS	All county performance tier
Ensemble	0.058	1
CDC-HistNB	0.059	2
USC-INLA	0.059	2
hybrid-hybrid	0.059	2
LANL-NBandP	0.059	2
MSSM-WED	0.061	2
Datart-PoissonFE	0.070	3
AMbeRland-RandomForest_anomaly	0.082	4
CDC-NaiveHist	0.088	4
FINforWN-MCMaWN	0.089	4
Kansas–Bayesian	0.126	5

We also examined forecast scores for a small subset of high-caseload counties (*n* = 49) that collectively account for ~50% of historical WNND caseload (county list in Table S2; results in Fig. S2, Table S3) to assess forecast skill in areas highly impacted by WNV where skillful forecasts could be particularly valuable. For these counties, WIS values were higher in general and we only identified three tiers of performance on the basis of bootstrap comparisons. Models that performed well within the all-county subset largely performed well for high-caseload counties; while the ordinal ranking of the six forecast models with the highest skill reshuffled all remained within the top two tiers of model performance. The ensemble model again demonstrated the highest skill for this subset, but the top performance tier also included five team-submitted models and the historical negative binomial baseline model. Of note, one team-submitted model (Kansas–Bayesian) performed substantially better in the high-caseload subset than in the all-county subset relative to other models due to more precise forecasts for the high-caseload counties.

Finally, we examined forecast skill in two more categories: counties that had or had not ever reported cases (2005–2021). While model performance for the counties with historical cases largely mirrored the all-county results, relative model performance reordered when we scored counties without historical cases, with three submitted forecasts marginally outperforming the ensemble forecast, though these differences were not statistically significant (Figs. S3, S4; Table S2).

### Comparing WIS and logarithmic scoring

**Fig. 4:**
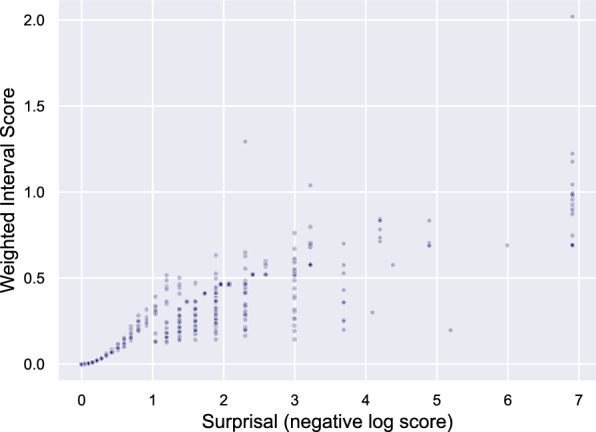
Comparison of scoring metrics. Scatter plot of surprisal (negative logarithmic score) versus weighted interval score of the ensemble forecast for each county (darker purple shading indicates overlapping points). Vertical alignment of points is an artifact of integer-based inputs into the calculation of logarithmic scores

We found a high Pearson correlation (*r* = 0.93) between surprisal (negative logarithmic score) and WIS for the ensemble model (Fig. 4). While the precise model ranking shifted when comparing model performance scores across score metrics, the tiers did not show large differences (Fig. S5 and Table S4).

### Model characteristics analysis

For forecasts across all counties, we found that forecast models using Bayesian methods or regression frameworks had higher average skill while models incorporating climate, demographic, or avian species data had worse skill. When examining the same characteristics of forecasts for high-caseload counties only, models incorporating mosquito surveillance and avian species data had higher skill, while those using climate or demographic data had worse performance. Regression results for counties with historical cases largely paralleled all-county results, but results for counties without historical cases showed reduced forecast skill for models using most of the data inputs under consideration (Table 2).

**Table 2:** Impact of model characteristics across county subsets

County subset	Covariate	2.5th percentile	median	97.5th percentile
(a) All counties	Bayesian	−0.020	−0.015	−0.009
	Regression	−0.013	−0.010	−0.006
	Climate	0.015	0.022	0.029
	Mosquito surveillance	0.009	0.021	0.038
	Demographic	0.018	0.029	0.045
	Any avian	0.009	0.015	0.022
(b) High caseload counties	Climate	0.083	0.175	0.267
	Mosquito surveillance	−0.151	−0.102	-0.045
	Demographic	0.265	0.544	1.270
	Any avian	−0.199	−0.138	−0.085
(c) Counties with historical cases	Regression	−0.023	−0.018	−0.014
	Climate	0.034	0.043	0.053
	Demographic	0.015	0.025	0.037
	Any avian	0.001	0.008	0.018
(d) Counties without historical cases	Climate	0.001	0.002	0.003
	Mosquito surveillance	0.002	0.003	0.005
	Demographic	0.002	0.003	0.004
	Any avian	0.002	0.003	0.003

Regression coefficients of individual model characteristics on weighted interval scores for models incorporating that model characteristic compared with the models that did not, determined by a Bayesian generalized linear model. Negative values indicate higher skill when the characteristic is included and positive values indicate lower skill when the characteristic is included. The median value of impact of these characteristics is shown, along with 95% confidence interval bounds. Analysis performed using (a) all counties (*n* = 3108), (b) high-caseload counties (*n* = 49), (c) counties with historical cases (*n* = 2054), and (d) counties without historical cases (*n* = 1054). Only covariates with significant coefficients included.

### County-contextual factors

**Fig. 5:**
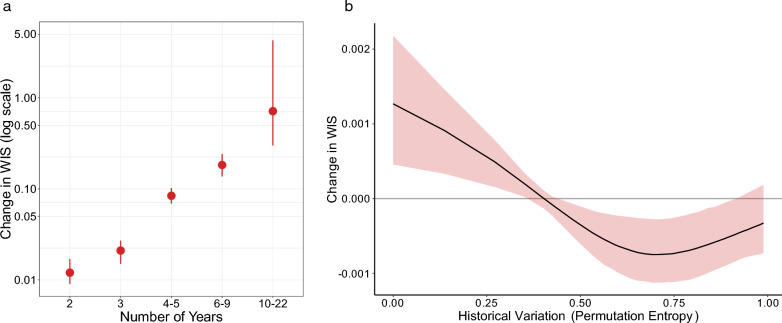
Influence of place-based contextual factors on ensemble forecast skill. **a** The response of forecast skill (WIS) to the binned number of years a county has reported WNND cases. Values represent the difference in WIS for each bin compared with a 1-year bin. Filled circle represents the median change while the lines represent the 95% confidence interval. **b** The response of forecast skill (WIS) to permutation entropy. Red shading represents the 95% confidence interval. Note the difference in *y*-axis scales

Our analysis of county contextual factors focused on three groupings of factors: environmental, demographic, and historical WNND. Associations between WIS and individual factors were analyzed, which revealed greater ensemble forecast skill (lower WIS) at both the lower and upper ends of the ranges for proportion urbanized, population > 65 years old, and total population size (Fig. S6). Skill was lower (higher WIS) at moderate levels of each of these variables. The opposite was true for mean minimum winter temperatures, with the highest skill at moderate temperatures and reduced skill at the lower and upper ends of the range. Ensemble forecast skill was highest for counties with the fewest historical years with WNND cases and declined (higher WIS) for counties with more prior years with reported WNND cases. Skill also declined for counties with greater permutation entropy, which was indicative of more volatility in historical WNND case numbers. When all factors of interest were combined within a multiple regression with backward selection, we found that only the number of years the county has reported WNND and permutation entropy were significantly associated with variation in skill (Fig. 5). Contextualized within this multiple regression, counties that had reported fewer years with WNND cases coincided with lower WIS (better skill), while counties with more years reporting WNND generally had worse forecast skill (Fig. 5a). Moreover, locations with low permutation entropy coincided with higher WIS (worse skill) and locations with high permutation entropy coincided with lower WIS (better skill; Fig. 5b).

## Discussion

### Ensemble performance

The ensemble forecast possessed the highest skill of all models and baselines over the forecast period and was statistically better than the historical negative binomial baseline skill, which was the forecast model with the highest skill in the 2020 Forecasting Challenge [11] and a performance benchmark (note that the ensemble forecast tied for the highest skill of all models in logarithmic scoring, the scoring metric used in the 2020 Challenge, although our analysis demonstrated WIS and logarithmic scoring produced broadly similar findings). Given that the ensemble did not outperform the historical negative binomial baseline in the 2020 Forecasting Challenge, this result is encouraging and may be a sign of increasing community skill in forecasting WNND. As in the Aedes Forecasting Challenge [20] and forecasts for COVID-19 [21], we used a median ensemble, which limits the impact of outlier forecasts that deviate from the larger suite of models. In other infectious disease forecasting efforts, some team forecasts have outperformed similar mean- or median-based ensemble forecasts, though ensembles have consistently been among the most skilled forecasts regardless of the specifics of their construction [21–24]. Given the success of the ensemble model, future WNV forecast model skill may be improved through the development of multi-model ensembles. Similarly, decision-makers may benefit from focusing on ensemble forecasts compared with individual forecasts when available; even individual forecasts which outperform an ensemble can only be identified retrospectively.

### Model characteristic analysis

Analyzing the importance of model components revealed a number of factors related to performance. First, in our all-county analysis, we found that models using a Bayesian regression framework or those using any regression framework (i.e., Bayesian or not) had higher skill (Table 2; note that results for the any regression and Bayesian regression frameworks are likely conflated as four of the six models with a regression framework had Bayesian components). Previous forecasting challenges for other diseases have also shown that statistical models often outperform dynamical models, perhaps related to not making specific assumptions about transmission dynamics, which could lead to overfitting and to their specific consideration of uncertainty [22, 23]. We also found that models utilizing climate and demographic data demonstrated reduced skill compared with those that did not, in opposition to the findings of the 2020 Challenge (see additional discussion on this discrepancy in *Differences with 2020 Challenge results* below).

Despite results here that indicate that including model covariates such as mosquito or climate data reduces forecast skill, these data have clear biological importance to WNV transmission and decreased performance may be due to overfitting or data limitations in spatiotemporal resolution, availability, and precision. Future forecast model developments should carefully consider these limitations when attempting to incorporate these variables. Regression model limitations appear to be particularly prominent for the 1041 counties (approximately 33%) that have not reported WNND historically. In a given year, only a small number of these counties (13 or 1.2% of this subset of counties in 2022) typically report their first case of WNND, a rare but important event that is difficult to predict and that may limit the benefits of regression models derived on this subset of counties. Moreover, future evaluation frameworks focusing on locations highly impacted by WNV may produce actionable insights on predictive factors in WNV susceptible areas.

These findings represent important considerations for forecasting but should be treated with caution. Each model was unique in multiple ways, thus we were unable to directly assess the specific impacts of each particular model characteristic. For example, we were not able to directly compare a model when it incorporated demographic data against the same model without demographic data. Thus, a poorly calibrated regression model that included climate data could have made it appear that inclusion of climate data negatively impacted forecast skill, even if the use of climate data actually improved skill for that model. This potential pitfall is exacerbated by a limited sample size of available models. While we attempted to limit the influence of outlying models by only including model characteristics that were present in a minimum of two models (see Text S4, Table S1), the relatively small number of total models (*n* = 9) limits such subsetting. Finally, we note that the way in which data was incorporated varied widely (e.g., “climate data” could include national average winter temperature or county-level March precipitation). A direct experimental comparison of forecasts from a single well-calibrated model can provide more insight on key data (e.g., [25]). However, different model structures and assumptions are also likely critical as shown here, necessitating larger comparative studies.

### County contextual factor analysis

Though six county-contextual factors proved significant in univariate regression models (Fig. S6), the two predominant county-contextual factors were the number of years a county had previously reported WNND and permutation entropy (Fig. 5), as determined via a multiple regression with backward selection. These two factors are highly correlated (*r* = 0.86) and individual univariate regressions (Fig. S6) of both factors showed that a higher number of years with reported WNND cases and increased entropy were both associated with increased WIS. These associations are unsurprising as counties with more years of reported cases have more complex patterns in year-over-year case counts (i.e., higher entropy), and are more difficult to predict. Both the number of years and entropy factors are correlated with county case counts and higher WIS values are associated with higher observations, a finding we also saw here despite log transforming forecasts and observations prior to scoring.

When combined in a multiple regression, the impact of the number of years of reported WNND strongly outweighed that of entropy by roughly three orders of magnitude. Additionally, the direction of the relationship between forecast skill and entropy inverted in the multiple regression compared with the univariate regression (contrast Fig. 5b—multivariate results, with Figure S6f—univariate results), and the magnitude of the relationship was much smaller. More years with cases creates more opportunity for higher entropy, thus these variables are correlated and the specific coefficients should therefore be interpreted with caution. Nonetheless, the overall associations were clear: by any metric, forecasting was less skilled for counties with more years with reported WNND cases.

This differed slightly from the findings in the 2020 Challenge in which total population, minimum extreme winter temperature, and permutation entropy were all associated with decreased forecast skill [11], though these findings do overlap with the findings of the univariate analysis performed here. The number of years of previously reported WNND may simply be a good combined indicator of other more foundational factors (e.g., population, minimum extreme winter temperature, baseline WNND rates).

### Differences with 2020 challenge results

Several findings differed between the 2020 [11] and 2022 Challenges. A number of factors may cause these discrepancies. First, both the forecasts submitted to the 2020 and 2022 Challenges and the sets of modeling teams were not identical. As a result, the method of incorporation of particular model characteristics may have varied. Second, the 2020 Challenge used a mean ensemble while we used a median ensemble, which was less subject to outlying forecasts. Third, the reported county-level cases of WNND naturally differed between the two years. While both years had fewer reported cases than historical 10-year medians, there were still nearly 50% more WNND cases reported in 2022 (827) than in 2020 (559), and the location of cases differed. These year-over-year differences may result in a given model scoring better simply by chance, independent of model construction. Fourth, there may be small differences in results created by using weighted interval scores instead of surprisal as the primary basis for comparison. Finally, changes in human behavior in 2020 because of the COVID-19 pandemic likely influenced differences in exposure to WNV vectors during the 2 years examined [26].

## Conclusions

In contrast to the 2020 WNV Forecasting Challenge, the ensemble forecast skill was statistically better than the historical negative binomial benchmark, though the raw difference in skill was marginal. This finding indicates that despite the wide-ranging skill of individual models to forecast WNV—most of which performed worse than the historical negative binomial baseline—the collective skill went beyond simply capturing the historical variability in case numbers. The ensemble forecasts predicted median or below median case numbers for 99% counties in 2022 in expectation of a relatively low WNV season. While 8% of counties actually reported case numbers above the county-level median, the national case counts were approximately 40% below the 10-year median count.

In both WNV Forecasting Challenges (2020, 2022), the results have revealed obstacles to identifying forecasts that are skillful beyond simple historical distributions. However, they have also resulted in insights on approaches that may drive WNV forecasting advances. First, the addition of real-time data, including case data, surveillance data, and environmental data, could greatly enhance efforts for within-season modeling efforts [27] by providing additional information relevant to transmission dynamics that is often not available in long lead-time forecasting challenges such as the one described here. Given the nature of the data involved, this would likely necessitate a more focal approach built upon partnerships with state or local agencies. Indeed, despite mixed efforts forecasting WNV at the national level, there are some examples of informative WNV forecasts and guidance tools at the local level [28–32], as well as encouraging efforts to predict infectious mosquitoes at fine spatial scales [33, 34]. These studies highlight the importance of local WNV ecology that may be difficult to capture in nation-wide modeling approaches; region-specific models, particularly models developed using ecologically-meaningful regions as opposed to geopolitical boundaries [35], may provide better avenues to capturing ecological dynamics by boosting the statistical signal of infrequently occurring outcomes across areas with relatively homogeneous large-scale dynamics. This coincides with the potential to grow our knowledge regarding the impacts of relevant covariates (e.g., environmental conditions) on WNV at higher levels of aggregation, though knowledge gained at these scales come with tradeoffs in their applicability. For example, state-level forecasts may stabilize predictions by reducing the number of zeros, but have limited practical value for public health and vector control actions.

However, while it may be possible to improve national or regional WNV forecasts, the utility of forecasts remains highest at the local scale. A remaining challenge here is that only ~10% of counties report WNND cases in a typical year, meaning that most forecasts accurately predict zero cases, and correctly forecasting zeros is a large proportion of forecasting scoring, even though the non-zeros are the bigger public health challenge. Considering counties with no, low, intermediate, or high case numbers separately may also be fruitful and improve interpretability of results and focus modeling efforts on the different public health needs of these counties. Local forecasts in high incidence areas with better data availability could drive effective local public health actions. In addition to potential gains through careful consideration of locations of focus and spatiotemporal scales of aggregation, machine learning techniques have also shown promise within disease forecasting broadly (e.g., [36, 37]), though they have yet to successfully outperform historical baseline models for WNV forecasting [25]. We encourage future WNV forecasting efforts, whether national, subnational, or local in nature, to pursue these potential pathways forward in their modeling efforts to reduce WNND burden and meet the CDC’s Division of Vector-Borne Diseases goal of reducing WNND to fewer than 500 annual cases by 2035 [38].

## Supplementary Information

Additional file 1. Supplementary Information for Evaluation of the 2022 West Nile Virus Forecasting Challenge. Appendix. Text S1: Participating Team Modeling Approaches. Text S2: Calculating Logarithmic Scores from Quantile Forecasts. Text S3: *P*-value Determination via Bootstrapping. Text S4: Model Covariate Factor Analysis. Table S1: Frameworks and Covariates Used in Model Development. Text S5: County-Specific Contextual Factor Analysis. Figure S1: Model-Specific Forecast Skill by Submission Month. Table S2: High-Caseload Counties. Table S3: Mean Model Scores for High Caseload and Counties with/without Historical Cases. Figure S2: Comparison of Mean Model Scores for High-Caseload Counties. Figure S3: Comparison of Mean Model Scores for Counties with Historical WNND Cases. Figure S4: Comparison of Mean Model Scores for Counties without Historical Caseload. Table S4: All County Mean Model Scores with Logarithmic Scoring. Figure S5: Comparison of Mean Model Scores for All Counties with Logarithmic Scoring. Figure S6: Influence of Place-Based Contextual Factors on Ensemble Forecast Skill.

## Data Availability

CDC ArboNET data for WNV cases are publicly available at https://www.cdc.gov/west-nile-virus/data-maps/historic-data.html. The datasets used for this study, as well as analysis coding scripts, are available in the WNV-forecast-data-2022 GitHub repository, https://github.com/cdcepi/WNV-forecast-data-2022/.

## Abbreviations

WNV: West Nile virus
WNND: West Nile virus neuroinvasive disease
CDC: U.S. centers for disease control and prevention
WIS: Weighted interval score
