# Supplementary material for: Evaluation of the 2022 West Nile virus forecasting challenge, USA
Source: Parasit Vectors. 2025 Apr 23;18:152. doi: 10.1186/s13071-025-06767-2 (PMC12020065; doi:10.1186/s13071-025-06767-2)
Supplement: Supplementary file 1 — Additional file 1. Supplementary Information for Evaluation of the 2022 West Nile Virus Forecasting Challenge. Appendix. Text S1: Participating Team Modeling Approaches. Text S2: Calculating Logarithmic Scores from Quantile Forecasts. Text S3: P-value Determination via Bootstrapping. Text S4: Model Covariate Factor Analysis. Table S1: Frameworks and Covariates Used in Model Development. Text S5: County-Specific Contextual Factor Analysis. Figure S1: Model-Specific Forecast Skill by Submission Month. Table S2: High-Caseload Counties. Table S3: Mean Model Scores for High Caseload and Counties with/without Historical Cases. Figure S2: Comparison of Mean Model Scores for High-Caseload Counties. Figure S3: Comparison of Mean Model Scores for Counties with Historical WNND Cases. Figure S4: Comparison of Mean Model Scores for Counties without Historical Caseload. Table S4: All County Mean Model Scores with Logarithmic Scoring. Figure S5: Comparison of Mean Model Scores for All Counties with Logarithmic Scoring. Figure S6: Influence of Place-Based Contextual Factors on Ensemble Forecast Skill. [file 13071_2025_6767_MOESM1_ESM.docx]

**Supplementary Information for Evaluation of the 2022 West Nile Virus Forecasting Challenge, United States**

This file contains: Text S1-S5, Tables S1-S4, Figures S1-S6.

**Text S1: Participating Team Modeling Approaches.** This supplemental text details the self-described methodology of each submitted forecasting model (primary affiliation). In each description below, “methods” provides a short (<200 character) summary of the methods with the (optional) “methods_long” section providing a fuller description. “Data_inputs” indicates any additional data sources beyond historical case data included in the model. Note that the descriptions here represent the final version submitted by teams; five teams (indicated with * below) updated these descriptions throughout the Challenge. These descriptions, along with additional information, can be found in the Challenge GitHub repository [1].

**AMbeRland-RandomForest_anomaly (University of Cambridge)**

***methods***: Random forest predictions based on historical temperature anomaly data.

***data_inputs***: NOAA GlobalTemp dataset of temperature anomalies.

***methods_long***: For each location, we used center coordinates to extract values of monthly temperature anomalies. These were used as covariates, and case numbers were used as predictors when training random forest model. For prediction, we bootstrapped historical values of monthly temperature anomalies for each location as predictors for 2022.

**Datart-PoissonFE (Binghamton University)**

***methods***: For this challenge, we used a fixed-effect Poisson regression model to fit our model using temperature, precipitation, last year mean cases of neighbors and past two years' cumulative cases.

***data_inputs***: Temperature which is the average temperature for each county per year (2000 to 2022, same years for the forecast) during each month (from ncdc), Precipitation which is the average precipitation for each county per year (2000 to 2022, same years for the forecast) during each month (from ncdc), Mean cases of each county neighbors for the previous year, Cumulative cases of each county for the past two years.

**FINforWN-MCMaWN (Indiana University)***

***methods***: Modulation function for the estimate of the mosquito relative density combined with the probability of observing West Nile transmission events assessed throughout a logistic regression model

***data_inputs***: Monthly precipitation and temperature records were retrieved from the WorldClim database at a spatial resolution of 30 seconds. Specifically, these records are the average for the years 1970-2000. Data on the human density were retrieved from the JRC Earth Observation Data and Processing Platform at a spatial resolution of 250 m x 250 m. We use mosquito capture data collected in different locations of the US.

***methods_long***: We assess the probability of observing West Nile transmission events by applying an ensemble of nested logistic regression models to records of at least two neuroinvasive cases for the years 2000-2021. The considered explanatory variables include the annual mean temperature, the annual mean precipitation, the maximum of monthly mean temperatures, the maximum of monthly mean precipitations, and the human density. We approximate the seasonal patterns characterizing the mosquito density with a modulation function of local temperature and precipitation records, by assuming that an increase in the adult abundance occurs as a consequence of persisting favorable conditions over a certain period. The model is informed with adult mosquito collection data across different times and locations of the US. Once calibrated, the model produces daily estimates of the relative abundance of female adults in any geographical area of interest. Obtained estimates were combined with the probability of WNV occurrence to estimate the expected cumulative number of neuroinvasive cases in each US county at the end of 2022. In particular, we assume that in the counties reporting sustained transmission, the number of WNV cases is proportional to the expected maximum number of mosquitoes. The model is calibrated through an MCMC approach applied to the negative binomial likelihood of observing the actual cumulative number of cases observed between 2012 and 2021. For the forecast of July, the logistic regression model is used to estimate the probability that the disease is endemic across the different counties. Specifically, the disease is classified as “endemic” in a county if at least one neuroinvasive case was reported in 3 different years between 2017 and 2021. The disease is marked as “non-endemic” in a county if no neuroinvasive cases were observed between 2000 and 2021. Counties where the disease is neither classified as “endemic” nor as “non-endemic”, are discarded from the analysis. Finally, the number of WNV neuroinvasive cases in a county is modeled as proportional to the estimated annual mean number of mosquitoes through a region-dependent parameter, which depends on the biome of the county under study. Model calibration is performed by considering only counties reporting 10 cumulative WNV cases between 2000 and 2021.

**LANL-NBandP (Los Alamos National Laboratory)***

***methods***: We divided our analysis between counties with less than 10 cases per year on average from 2005-2021 and more. Then, we used historical case data and climate data as predictor variables and applied a negative binomial or poisson distribution.

***data_inputs***: 2005-2021 neuroinvasive case data, PRISM Climate Data from Oregon State University

***methods_long***: We employed three different approaches for our model. We first analyzed the mean annual case counts from 2005-2021 for each county. If the county had equal to or less than 10 cases per year on average, we used the annual WNND case data from 2005-2021 and applied a negative binomial or Poisson distribution based on the historical WNND case counts. Where variance of the annual case counts was greater than the mean, we applied a negative binomial distribution calculated from the historical data. If the variance of the annual case counts was less than the mean, we applied a Poisson distribution. For counties that had greater than 10 cases per year on average (n = 13), we created a linear or quadratic forecast model of mean annual case incidence (cases per 100,000 population per year, using county-level population data). We used the single best predictor variable from annual or seasonal (SON previous year, DJF starting previous year, MAM of same year) mean air temperature, maximum air temperature, minimum air temperature, mean dew point temperature, mean maximum vapor pressure deficit, mean minimum vapor pressure deficit, cumulative precipitation, or WNND case counts of the previous year. All climate variables were processed from the PRISM Climate Group at Oregon State University. For future 2022 data, we used the provisional 6 month PRISM climate data available. We created a point forecast to represent the 50% percentile using the single-predictor models. Then, we combined this point forecast with the historical WNND case counts for each of the counties to create a negative binomial distribution.

**MSSM-WED (Mount Sinai School of Medicine)**

***methods***: Here we use a hierarchical structure to combine the local and state distributions together. The local level gets 80% of the weight while the state gets 20%.

***data_inputs:*** 2003 - 2021 neuroinvasive case data

***methods_long***: Here we use a hierarchical structure to combine the local, county-level empirical historical neuroinvasive human case data, with regional, empirical distribution of all county level outbreaks within the state to develop an optimized weighted distribution. Simulations were run to validate the optimal weighting using a leave one year out approach from 2003 to 2021. The optimal weights were 80% to the local level empirical distribution and 20% to the state distribution.

**USC-INLA (University of South Carolina)***

***methods***: County-Level Bayesian Negative Binomial regression with a log-link function using R-INLA

***data_inputs***: Land cover data were obtained on the county scale from the national land cover database. Vector data were obtained through the Defense Technical Information Center, Population data was obtained through the US Census Bureau, and elevation data were obtained from the USGS DEM 100-Meter Resolution Elevation of the Conterminous United States, and bird species data were obtained from the eBird database

***methods_long***: Predictions were calculated using a Bayesian Negative Binomial regression with a log-link function. Predictors chosen for the final model included the population of individuals aged 65 and up in each county, open water, developed medium intensity, developed high intensity, barren land, deciduous forest, evergreen forest, mixed forest, shrub scrub, herbaceous, and woody wetlands land cover data, sparrow species count, jay species count, crow species count, time variables to account for oscillating cycles in West Nile Virus cases, and year. As West Nile Virus cases tend to exhibit a three-year cycle of increase and decrease, indicator variables for cycle-year (cycle year 1, cycle year 2, or cycle year 3 (reference level) were included in the model. Because the effect of time varied across regions, we used random effects to allow the coefficients for year to be county-specific, in addition to a random effects intercept. Specifically, these coefficients followed a conditional auto-regressive model at the county level. An integrated nested LaPlace approximation (INLA) model in R software was used to fit our model. This provided a faster way to fit our model than standard Markov chain Monte Carlo methods.

**hybrid-hybrid (University of Nebraska-Lincoln)***

***methods***: NB base prediction with FLM approach for Great Plains, custom model for Chicago area, Bayesian EAKF for NY and CT counties. Reported cases were used to set minimum values

ensemble_of_hub_models: false

***data_inputs***: FLM Forecast uses historical human cases, population, lagged precipitation, temperature, Standardized Precipitation Index, and Standardized Precipitation and Evapotranspiration Index. Chicago forecast uses historical human cases, historical minimum infection rates for mosquitoes, and present-year temperature and precipitation. EAKF model uses historical and current year mosquito abundance and infection status. All were adjusted based on cases reported to ArboNET (CDC) and New York State Department of Health.

***methods_long***: A Negative Binomial (NB) model was fit to every county in the US. Functional Linear Modeling (FLM) was used for Great Plains counties from the following states: Nebraska, Kansas, South Dakota, North Dakota, Texas, Oklahoma, Montana, Minnesota, Colorado, Wyoming, Iowa, and New Mexico. The Chicago model used previously published models to first predict minimum infection rates, and then used minimum infection rates to predict human cases. The EAKF was modified from DeFelice et al. 2017 to work with mosquito-only data for counties with at least one positive pool and sufficient data (Nassau and Rockland, New York State; New London, Fairfield, and Hartford, Connecticut). Further research is needed to adjust the weighting. The FLM predictions and the NB were combined into an ensemble, weighted based on differences in historical Continuous Ranked Probability Scores for both models. We did not have time to compute historical performance for the EAKF and Chicago models. The Chicago model was assigned 100% weight and the EAKF 50% in their respective domains. Finally, any quantiles that had fewer predicted human cases than had been reported on ArboNET for the year were adjusted to the minimum known number of human cases for that county. Starting in August, these were also adjusted based on numbers from NYC Department of Health, as these were higher than those in ArboNET.

**kansas-bayesian (USDA-Agricultural Research Service)***

***methods***: Bayesian hierarchical model.

***data_inputs***: Confirmed cases, Temperature, Precipitation, avian phylodiversity, US Census (population; income)

***methods_long***: A two-part Bayesian hierarchical model was developed. The model includes spatial and temporal effects as well as spatially varying coefficients (SVC) for climate factors to enable location-specific estimation of climatic influence. Avian host phylodiversity, virus surveillance, and economic variables were added as predictors.

**Text S2: Calculating Logarithmic Scores from Quantile Forecasts**

To generate a logarithmic score from a quantile forecast, we first estimated probability from an approximate cumulative distribution function. For observed values within forecast quantiles, we calculated the probability assigned to the outcome as the linear interpolation between the closest quantiles. For observed values outside the 1% and 99% quantiles, we assigned a value of 0.001 (i.e., 0.1%). The logarithmic score for each county was then calculated as the natural logarithm of this probability (i.e., log(p)).

**Text S3: *P-*value Determination via Bootstrapping**

We compared each pair of models by randomly selecting 3,108 county-level WIS scores with replacement 1,000 times, calculating the difference in average WIS for each bootstrap sample. We then calculated *P*-values of score differences across each combination of forecasts from the proportion of bootstrap samples for which a model had a lower WIS (i.e., better score) than the other. We assessed differences between models at three thresholds (p < 0.1, p < 0.05, p < 0.01), including a correction for family-wise multiple-comparison errors by the Holm-Bonferroni Method [2]. We then manually separated models into performance tiers reflecting the relative forecast skill and the bootstrapped significance analysis (Table 1 and Figure 2). To examine model skill specifically for locations highly impacted by WNV, we replicated this analysis for a subset of high-incidence counties (the 49 highest burden counties that collectively contribute 50% of historical national WNND caseload).

**Text S4: Model Covariate Factor Analysis**

*Model component regression modeling*

To facilitate comparison between the 2020 Challenge [3] and our analysis of the 2022 Challenge, we largely considered model characteristics examined in the 2020 Challenge (Table S1). Our initial list of modeling frameworks under consideration included mechanistic, ensemble, artificial intelligence (AI), Bayesian, and “any regression” (i.e., any model framework with a regression component, broadly defined). Our initial list of data inputs under consideration included WNV incidence, climate, mosquito surveillance, “any equine”, land use, “any mosquito” (i.e., mosquito surveillance or mosquito species presence/absence), and “any avian.” However, we narrow our model covariates to only analyze model characteristics with mixed model inclusion (i.e., included or excluded by a minimum of two models) in an attempt to constrain the influences of outlying models with our analysis (see Table S1 for illustration of model characteristic presence). This restriction limits our analysis to three modeling frameworks (ensemble, Bayesian, and “any regression” components) and four data inputs (climate, mosquito surveillance, demographic, and “any bird” data). Note that while we identified four teams using “any mosquito data”, it is not sufficiently distinct from the “mosquito surveillance data” categorization as only one model utilized mosquito data beyond mosquito surveillance. Thus, we did not include this categorization in our analyses.

**Table S1: Frameworks and Covariates Used in Model Development.** Classifications of model frameworks and covariates for each submitted and baseline model. Bottom row shows covariates included in final model covariate analysis. Complete names of abbreviated categorizations are: Mech. = Mechanistic, Ensem. = Ensemble, AI = Artificial Intelligence, Bayes. = Bayesian, Any Reg. = “Any Regression”, Hist. WNV = Historical WNV, Mosq. Surv. = Mosquito Surveillance, Demo. = Demographics, Any Mosq. = “Any Mosquito.”

|  | **Modeling Frameworks** | | | | | **Data Inputs** | | | | | | | |
| --- | --- | --- | --- | --- | --- | --- | --- | --- | --- | --- | --- | --- | --- |
|  | Mech. | Ensem. | AI | Bayes. | Any Reg. | Hist. WNV | Climate | Mosq. Surv. | Demo. | Any equine | Land use | Any Mosq. | Any Avian |
| CDC-HistNB | N | N | N | N | N | **Y** | N | N | N | N | N | N | N |
| hybrid-hybrid | **Y** | **Y** | N | **Y** | **Y** | **Y** | **Y** | **Y** | **Y** | N | N | **Y** | N |
| MSSM-WED | N | **Y** | N | N | N | **Y** | N | N | N | N | N | N | N |
| LANL-NBandP | N | N | N | N | **Y** | **Y** | **Y** | N | N | N | N | N | N |
| USC-INLA | N | N | N | **Y** | **Y** | **Y** | N | N | **Y** | N | **Y** | **Y** | **Y** |
| Datart-PoissonFE | N | N | N | N | **Y** | **Y** | **Y** | N | N | N | N | N | N |
| CDC-NaiveHist | N | N | N | N | N | **Y** | N | N | N | N | N | N | N |
| AMbeRland-RF_anomaly | N | N | **Y** | N | N | **Y** | **Y** | N | N | N | N | N | N |
| FINforWN-MCMaWN | N | **Y** | N | **Y** | **Y** | **Y** | **Y** | **Y** | **Y** | N | N | **Y** | N |
| kansas-bayesian | N | N | N | **Y** | **Y** | **Y** | **Y** | **Y** | **Y** | N | N | **Y** | **Y** |
| **Included in Analysis** | N | **Y** | N | **Y** | **Y** | N | **Y** | **Y** | **Y** | N | N | N | **Y** |

**Text S5: County-Specific Contextual Factor Analysis.**

We examined a wide range of county-specific factors categorized into 1) environmental factors, 2) human demographics, and 3) historical WNND case incidence. Environmental factors data were extracted and calculated from the U.S. Census Bureau [4] and National Land Cover Database data [5] and included: land area, water area, percent of land urbanized, percent of land cropland, percent of land wetlands, and mean extreme winter temperature. The demographics factors were extracted and calculated from the U.S. Census Bureau included: total population, population > 65 years old, percentage of total population > 65 years old, and population density. Historical WNND factors were calculated from CDC ArboNET data [6] and included: potential entropy [7], first year of reported WNND, is 2022 the first year with reported WNND, number of years with reported WNND, total reported WNND cases, median historical WNND caseload, mean historical WNND caseload, median historical WNND incidence, mean historical WNND incidence, and have WNND cases ever been reported.

We first fitted each county-contextual variable with a Bayesian generalized additive model for a number of differentially fit splines (2-10 knots) to determine the best fit for each factor. All factors within each of the three distinct factor types (environmental, demographic, and historical WNND) were then collectively compared to determine the factor within each type which most impacts location-specific forecast skill. The most salient factors were percent of land urbanized and mean extreme winter temperature (environmental), population > 65 years old and total population (demographic), and number of years with reported WNND and permutation entropy (historical WNND). These remaining six factors were then input into a generalized additive model where we performed backward selections to identify the contextual factors that most impact forecast skill.

**References**

1. GitHub - cdcepi/WNV-forecast-data-2022: Data and forecast submission repository for the 2022 CDC West Nile virus Forecasting Challenge. 2024. https://github.com/cdcepi/WNV-forecast-data-2022. Accessed 12 Sept 2024.
2. Holm S. A simple sequentially rejective multiple test procedure. Scandinavian journal of statistics. 1979 Jan 1:65-70.
3. Holcomb KM, Mathis S, Staples JE, Fischer M, Barker CM, Beard CB, et al. Evaluation of an open forecasting challenge to assess skill of West Nile virus neuroinvasive disease prediction. Parasites & Vectors. 2023 Jan 12;16(1):11.
4. U.S. Census Bureau. Gazetteer Files. Census.gov. 2024. https://www.census.gov/geographies/reference-files/time-series/geo/gazetteer-files.html. Accessed 12 Sept 2024.
5. Multi-resolution characteristics consortium. NLCD 2011 land cover (CONUS). 2011. Available from: https://www.mrlc.gov/data/nlcd-2011-land-cover-conus
6. West Nile Virus Historic Data (1999-2023). U.S. Centers for Disease Control and Prevention. 2024. https://www.cdc.gov/west-nile-virus/data-maps/historic-data.html. Accessed 12 Sept 2024.
7. Henry M, Judge G. Permutation entropy and information recovery in nonlinear dynamic economic time series. Econometrics. 2019 Mar 12;7(1):10.


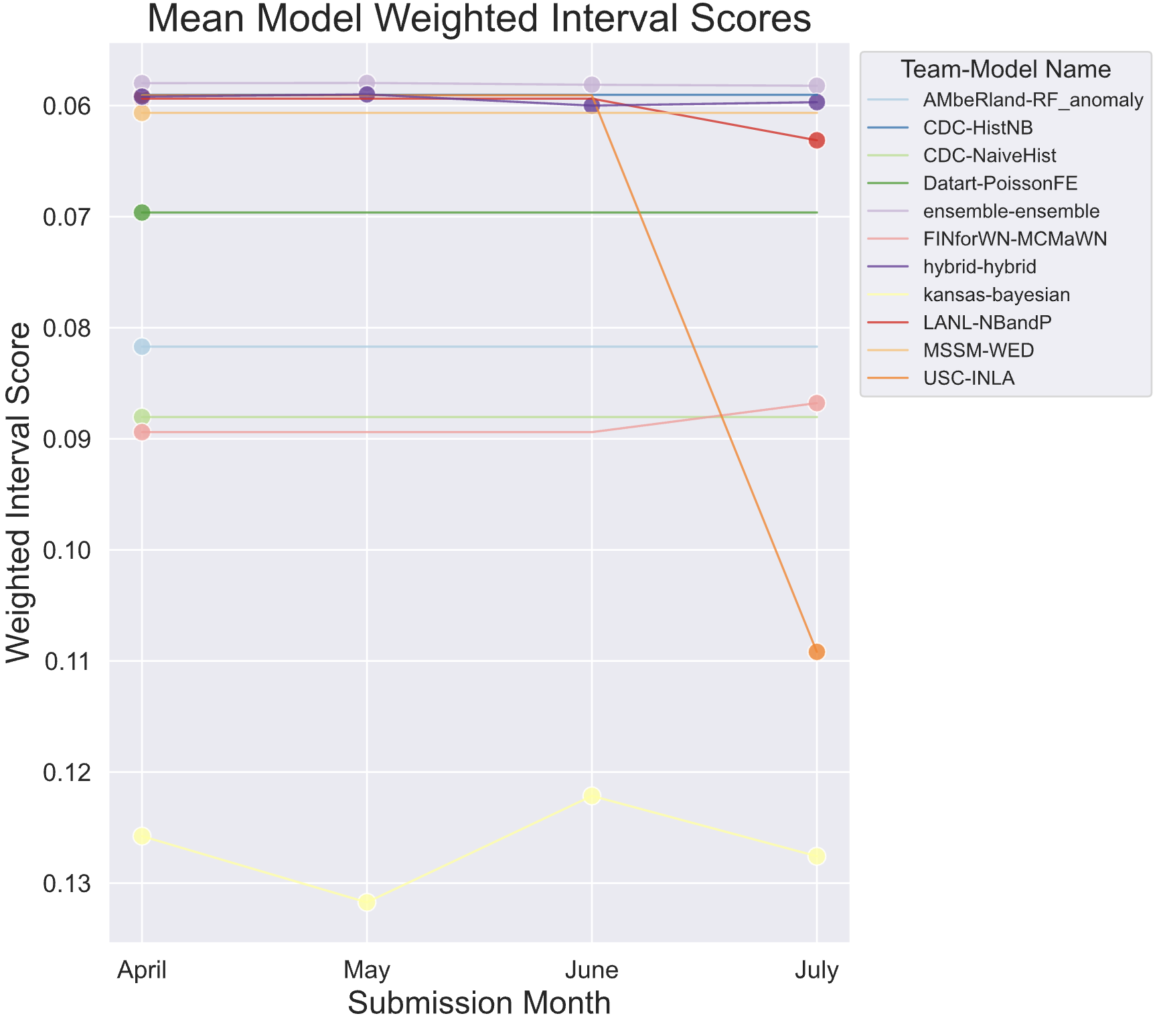


**Figure S1: Model-Specific Forecast Skill by Submission Month.** All-county mean forecast skill (WIS) for each submission month. Filled points represent WIS for forecasts submitted in the respective month; if a team did not submit a new forecast for a given month, the WIS of the latest submitted forecast is carried over to subsequent months as a horizontal line.

**Table S2: High-Caseload Counties.** This table lists the 49 highest WNND caseload counties that collectively contributed ~50% of total U.S. WNND caseload from 2005-2021.

^+^ Federal Information Process Standards (FIPS) code uniquely identifies the state (2-digit FIPS) and county (3-digit FIPS).

| **FIPS^+^** | **State-County** | **Total Historical WNND Cases** | **Historical WNND Cases (% of national caseload)** |
| --- | --- | --- | --- |
| 04013 | Arizona-Maricopa | 1,681 | 8.12 |
| 06037 | California-Los Angeles | 1,375 | 6.64 |
| 17031 | Illinois-Cook | 656 | 3.17 |
| 06059 | California-Orange | 467 | 2.26 |
| 48113 | Texas-Dallas | 420 | 2.03 |
| 48201 | Texas-Harris | 397 | 1.92 |
| 06065 | California-Riverside | 330 | 1.59 |
| 48439 | Texas-Tarrant | 296 | 1.43 |
| 26163 | Michigan-Wayne | 234 | 1.13 |
| 48141 | Texas-El Paso | 224 | 1.08 |
| 06029 | California-Kern | 219 | 1.06 |
| 04019 | Arizona-Pima | 213 | 1.03 |
| 06071 | California-San Bernardino | 213 | 1.03 |
| 06099 | California-Stanislaus | 198 | 0.96 |
| 06067 | California-Sacramento | 162 | 0.78 |
| 17043 | Illinois-DuPage | 148 | 0.72 |
| 06019 | California-Fresno | 147 | 0.71 |
| 06107 | California-Tulare | 144 | 0.70 |
| 36059 | New York-Nassau | 134 | 0.65 |
| 08123 | Colorado-Weld | 124 | 0.60 |
| 04021 | Arizona-Pinal | 120 | 0.58 |
| 28049 | Mississippi-Hinds | 108 | 0.52 |
| 31055 | Nebraska-Douglas | 106 | 0.51 |
| 48121 | Texas-Denton | 105 | 0.51 |
| 06073 | California-San Diego | 105 | 0.51 |
| 06007 | California-Butte | 100 | 0.48 |
| 22033 | Louisiana-East Baton Rouge | 99 | 0.48 |
| 22017 | Louisiana-Caddo | 99 | 0.48 |
| 40109 | Oklahoma-Oklahoma | 98 | 0.47 |
| 36081 | New York-Queens | 96 | 0.46 |
| 06077 | California-San Joaquin | 93 | 0.45 |
| 32003 | Nevada-Clark | 88 | 0.43 |
| 49035 | Utah-Salt Lake | 88 | 0.43 |
| 39035 | Ohio-Cuyahoga | 88 | 0.43 |
| 48339 | Texas-Montgomery | 87 | 0.42 |
| 48453 | Texas-Travis | 86 | 0.42 |
| 08069 | Colorado-Larimer | 83 | 0.40 |
| 40143 | Oklahoma-Tulsa | 82 | 0.40 |
| 08031 | Colorado-Denver | 80 | 0.39 |
| 26081 | Michigan-Kent | 80 | 0.39 |
| 47157 | Tennessee-Shelby | 79 | 0.38 |
| 48085 | Texas-Collin | 78 | 0.38 |
| 26099 | Michigan-Macomb | 77 | 0.37 |
| 08013 | Colorado-Boulder | 76 | 0.37 |
| 26125 | Michigan-Oakland | 73 | 0.35 |
| 42101 | Pennsylvania-Philadelphia | 71 | 0.34 |
| 36103 | New York-Suffolk | 71 | 0.34 |
| 08001 | Colorado-Adams | 70 | 0.34 |
| 48303 | Texas-Lubbock | 69 | 0.33 |
|  | **Total:** | 10,337 | 49.95 |

**Table S3: Mean Model Scores for High Caseload and Counties with/without Historical Cases.** Mean weighted interval scores across three subsets of counties in the contiguous United States, ordered by WIS across all counties (Table 2). The high caseload county subset consists of the 49 highest WNND caseload counties (Table S1). The subsets of counties with and without historical caseload are defined by the presence or absence of WNND cases for each county from 2005-2021. See Fig. S2-S4 for visual comparison of WIS for these three subsets.

| **Model** | **High-**  **Caseload WIS** | **High-**  **Caseload Tier** | **Counties With Historical Cases WIS** | **Counties With Historical Cases Tier** | **Counties Without Historical Cases WIS** | **Counties Without Historical Cases Tier** |
| --- | --- | --- | --- | --- | --- | --- |
| Ensemble | 0.432 | 1 | 0.091 | 1 | 0.011 | 1 |
| CDC-HistNB | 0.458 | 1 | 0.093 | 2 | 0.011 | 1 |
| USC-INLA | 0.512 | 1 | 0.093 | 2 | 0.011 | 1 |
| hybrid-hybrid | 0.450 | 1 | 0.093 | 2 | 0.011 | 1 |
| LANL-NBandP | 0.444 | 1 | 0.093 | 2 | 0.012 | 1 |
| MSSM-WED | 0.460 | 1 | 0.095 | 2 | 0.012 | 1 |
| Datart-PoissonFE | 0.735 | 2 | 0.111 | 3 | 0.011 | 1 |
| AMbeRland-RF_anomaly | 0.717 | 2 | 0.131 | 3 | 0.012 | 1 |
| CDC-NaiveHist | 1.359 | 3 | 0.135 | 4 | 0.022 | 2 |
| FINforWN-MCMaWN | 0.977 | 2 | 0.129 | 3 | 0.034 | 3 |
| kansas-bayesian | 0.511 | 1 | 0.148 | 4 | 0.094 | 4 |


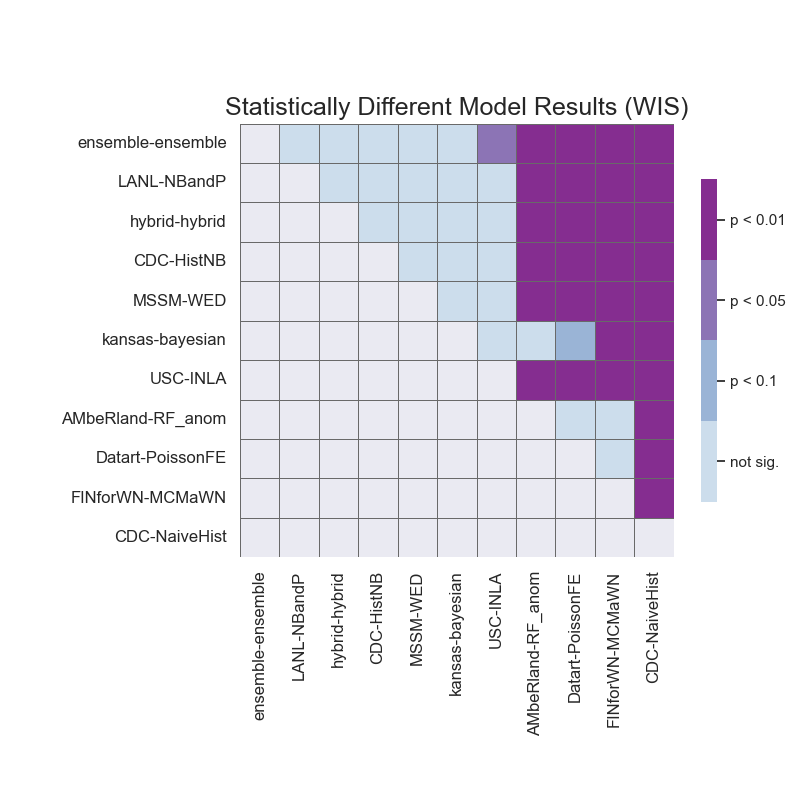


**Figure S2: Comparison of Mean Model Scores for High-Caseload Counties.** Level of statistical significance of differences in model performance is shown for each pair of models (blue-purple shading). Comparison performed using WIS for high caseload counties (Table S1) using bootstrapping methodology.


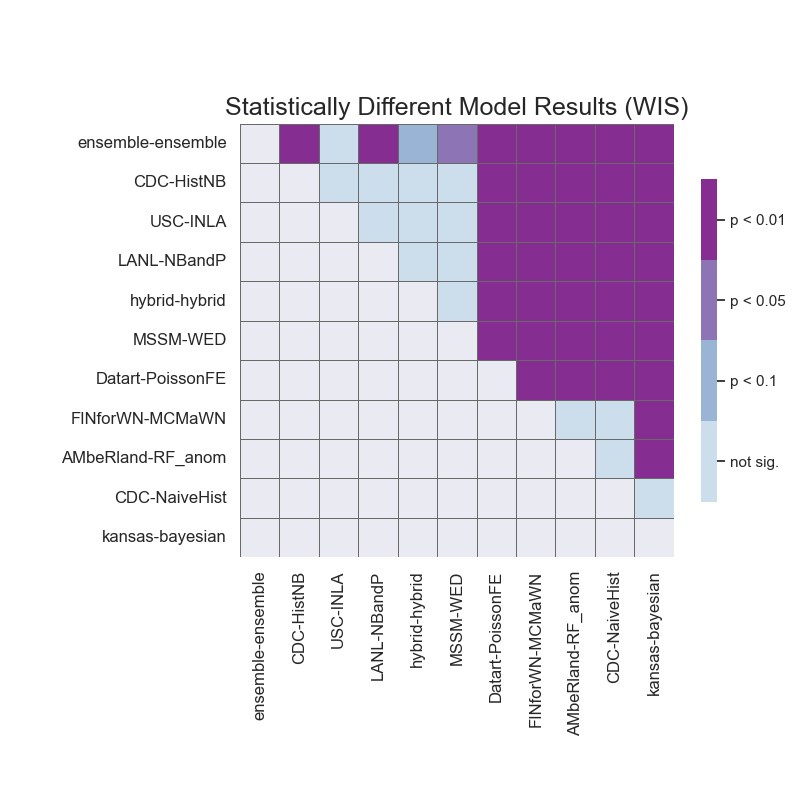


**Figure S3: Comparison of Mean Model Scores for Counties with Historical WNND Cases.** Level of statistical significance of differences in model performance is shown for each pair of models (blue-purple shading). Comparison performed using WIS for counties that have reported WNND historically using bootstrapping methodology.


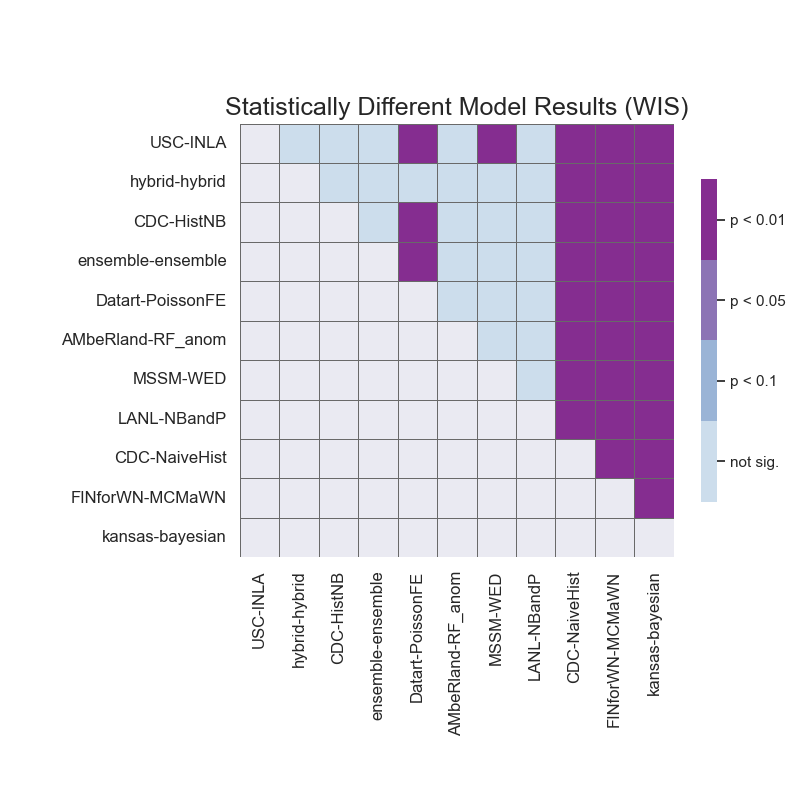


**Figure S4: Comparison of Mean Model Scores for Counties without Historical Caseload.** Level of statistical significance of differences in model performance is shown for each pair of models (blue-purple shading). Comparison performed using WIS for counties that have not reported WNND historically using bootstrapping methodology.

**Table S4: All County Mean Model Scores with Logarithmic Scoring.** Mean logarithmic scores across all counties in the contiguous U.S., ordered by model skill.

| **Model** | **All-County Logarithmic Score** | **All-County Performance Tier** |
| --- | --- | --- |
| USC-INLA | -0.376 | 1 |
| hybrid-hybrid | -0.376 | 1 |
| CDC-HistNB | -0.376 | 1 |
| ensemble-ensemble | -0.376 | 1 |
| LANL-NBandP | -0.387 | 2 |
| MSSM-WED | -0.400 | 2 |
| Datart-PoissonFE | -0.432 | 3 |
| CDC-NaiveHist | -0.478 | 4 |
| FINforWN-MCMaWN | -0.503 | 5 |
| AMbeRland-RandomForest_anomaly | -0.586 | 6 |
| kansas-bayesian | -0.707 | 7 |


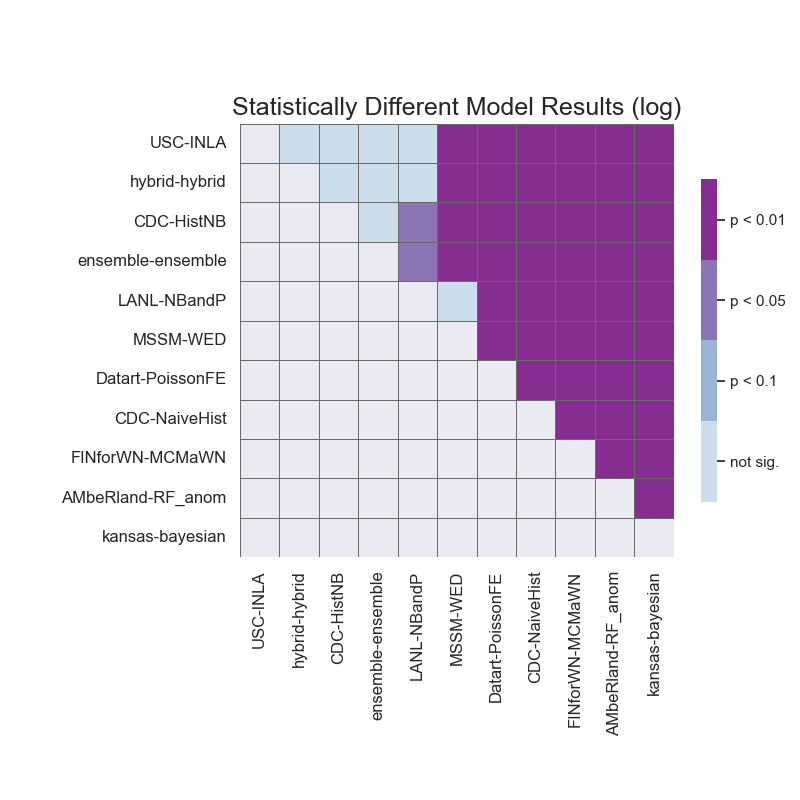


**Figure S5: Comparison of Mean Model Scores for All Counties with Logarithmic Scoring.** Level of statistical significance of differences in model performance for each pair of models (blue-purple shading). Comparison performed using logarithmic scoring for all counties using bootstrapping methodology.


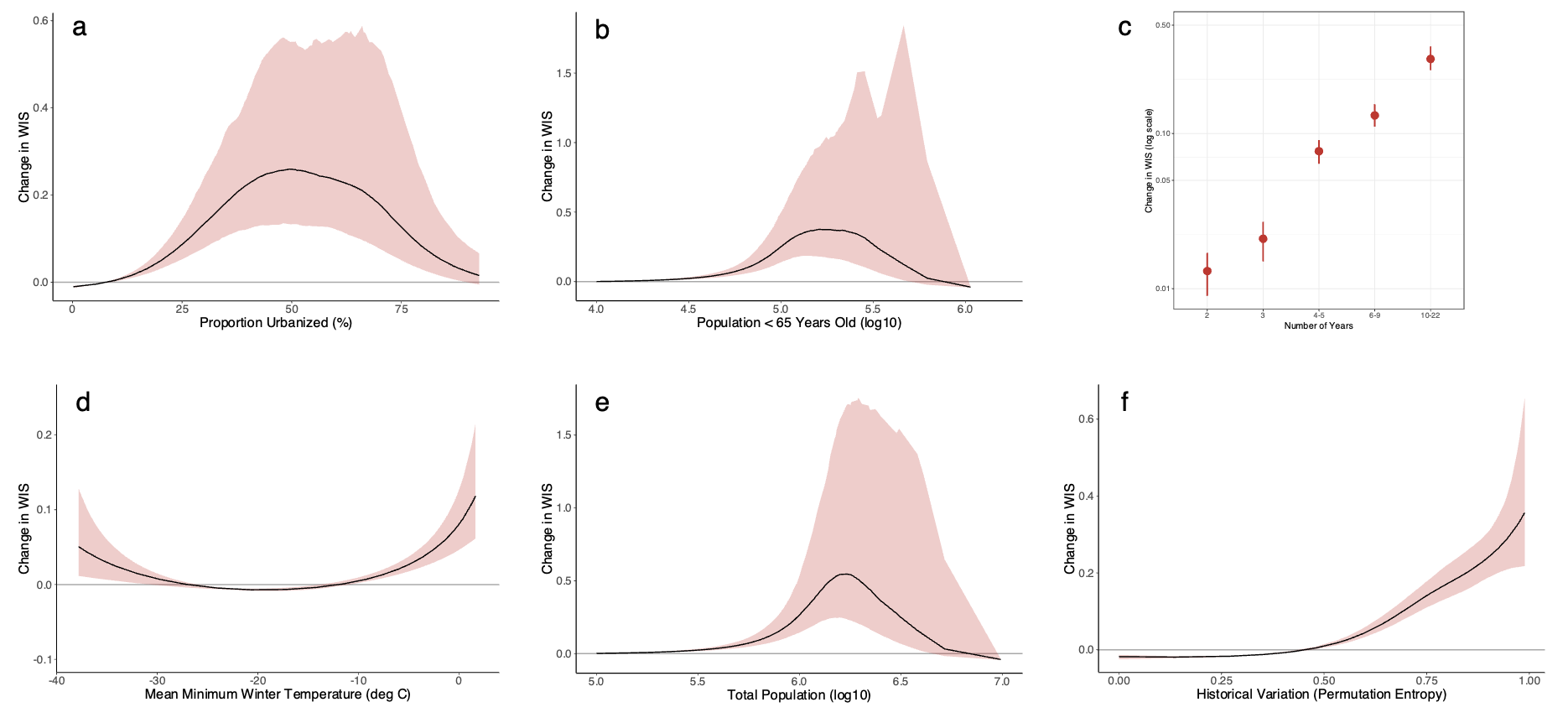


**Figure S6: Influence of Place-Based Contextual Factors on Ensemble Forecast Skill.** Ensemble forecast skill (WIS) in relation to a) the percentage of urbanization of a county, b) population over 65 years of age, c) number of years with reported WNND cases (relative to one year), d) mean extreme winter temperature, e) total population, and f) historical variation in WNND cases (permutation entropy). 95% confidence intervals indicated by red shading (a-b, d-f) or lines (c) around the median (black line or filled dot). In c), estimates represent the difference in WIS for each bin compared to a one year bin. Grey horizontal line indicates no change in WIS. Note the difference in y-axes scales across panels.
